# A selective screening program for the early detection of mucopolysaccharidosis: Results of the FIND project – a 2-year follow-up study

**DOI:** 10.1097/MD.0000000000006887

**Published:** 2017-05-12

**Authors:** Cristóbal Colón, J. Victor Alvarez, Cristina Castaño, Luís G. Gutierrez-Solana, Ana M. Marquez, María O’Callaghan, Félix Sánchez-Valverde, Carmen Yeste, María-Luz Couce

**Affiliations:** a Unit of Diagnosis and Treatment of Congenital Metabolic Diseases, Service of Neonatology, Department of Pediatrics, Hospital Clínico Universitario de Santiago, CIBERER, Health Research Institute of Santiago de Compostela (IDIS), Santiago de Compostela; b Service of Pediatrics, Hospital Universitario Infanta Leonor; c Section of Pediatric Neurology, Service of Pediatrics, Hospital Infantil Universitario Niño Jesús, CIBERER, Madrid; d Unit of Diagnosis and Treatment of Congenital Metabolic Diseases, Service of Pediatrics Gastroenterology, Department of Pediatrics, Hospital Materno Infantil de Badajoz; e Department of Neuropaediatrics, Hospital Sant Joan de Déu, Esplugues, Barcelona; f Gastroenterology and Paediatric Nutrition Unit, Hospital Virgen del Camino, Pamplona; g Department of Pediatrics, Hospital Costa del Sol de Marbella, Spain.

**Keywords:** enzymatic activity, glycosaminoglycans, mucopolysaccharidosis, screening, urine sample

## Abstract

The mucopolysaccharidoses (MPSs) are underdiagnosed but they are evaluated in few newborn screening programs, probably due to the many challenges remaining, such as the identification of late-onset phenotypes. Systematic screening at the onset of clinical symptoms could help to early identify patients who may benefit from specific treatments. The aim of this prospective study was to assess a novel selective screening program, the FIND project, targeting patients aged 0 to 16 years with clinical manifestations of MPS. The project was designed to increase awareness of these diseases among pediatricians and allow early diagnosis.

From July 2014 to June 2016, glycosaminoglycan (GAG) levels normalized to creatinine levels were determined in urine-impregnated analytical paper submitted by pediatricians who had patients with clinical signs and/or symptoms compatible with MPS. When high GAG concentrations were detected, a new liquid urine sample was requested to confirm and identify the GAG present. When a specific form of MPS was suspected, enzyme activity was analyzed using blood-impregnated paper to determine MPS type (I, IIIB, IIIC, IVA, IVB, VI, or VII). Age-specific reference values for GAG were previously established using 145 urine samples from healthy children.

GAG levels were normal in 147 (81.7%) of the 180 initial samples received. A liquid sample was requested for the other 33 cases (18.3%); GAG levels were normal in 13 of these and slightly elevated in 12, although the electrophoresis study showed no evidence of MPS. Elevated levels with corresponding low enzymatic activity were confirmed in 8 cases. The mean time from onset of clinical symptoms to detection of MPS was 22 months, and just 2 cases were detected at the beginning of the project were detected with 35 and 71 months of evolution of clinical symptoms. Our screening strategy for MPS had a sensitivity of 100%, a specificity of 85%, and a positive predictive value of 24%.

The FIND project is a useful and cost-effective screening method for increasing awareness of MPS among pediatricians and enabling the detection of MPS at onset of clinical symptoms.

## 1 Introduction

The mucopolysaccharidoses (MPSs) are a group of inherited metabolic diseases caused by defects in the lysosomal hydrolytic enzymes needed to break down glycosaminoglycans (GAGs).^[1]^ These defects result in the accumulation of these macromolecules in the cells of various organs, causing progressive multisystemic lesions. The overall incidence of MPS is over 1 case per 25,000 live births.^[2]^ The incidence, however, of the different forms of MPS varies.^[3–6]^ Eleven enzyme deficiencies have been identified to date, and some of the MPS types have been further divided into subtypes according to the enzyme defect involved and the type of GAG eliminated in the urine (chondroitin sulfate [CS], dermatan sulfate [DS], heparan sulfate [HS], keratan sulfate [KS], and/or hyaluronic acid). Although certain forms of MPS may exhibit a characteristic Hurler phenotype, they all share some very similar clinical characteristics.^[3,6,7]^

Diagnosis is challenging as the MPS is not generally among the 1st conditions considered by pediatricians, family doctors, internists, or other health professionals in the differential diagnosis. Delays in diagnosis are thus very common,^[8,9]^ especially as the varying signs and symptoms are frequently perceived as being independent of each other and are thus treated separately. Furthermore, certain attenuated forms of MPS are characterized by delayed presentation and nonspecific symptoms, causing confusion with other diseases and further complicating diagnosis.^[10]^

Treatment of MPS has advanced significantly in recent years with the use of enzyme replacement therapy (ERT) and hematopoietic stem cell transplantation to prevent or treat progression.^[11–15]^ Other therapeutic approaches, such as chemical chaperone therapy,^[16]^ substrate reduction therapy, gene therapy,^[17]^ and stop-codon read-through,^[18]^ have been tested in clinical trials or are currently under development. Breakthroughs in treatment can have an important impact on prognosis and quality of life for many patients.^[19]^ Given the progressive nature of MPS, early diagnosis and treatment is crucial.^[20]^ Although some lysosomal storage disorders (LSDs) are now considered candidates for newborn screening programs, many challenges remain, such as the identification of late-onset phenotypes.^[21]^ As a result, very few newborn screening programmes around the world include LSDs.

Given the progressive nature of MPS and the importance of early diagnosis, it is essential to provide the various specialists who come into contact with these patients with information and tools that will prompt them to consider MPS in the differential diagnosis. The aim of the study was to test a selective screening programme aimed at increasing the early diagnosis of MPS by improving awareness of these disorders among pediatricians. To this end, we designed a novel selective screening strategy involving the assessment of GAGs in urine samples taken from an at-risk population with signs and/or symptoms. The program was launched in Spain in 2014.

## 2 Methods

### 2.1 Study design

We conducted a prospective study to evaluate a novel selective screening strategy for patients with suspected MPS. On July 1, 2014, the Unit for the Diagnosis and Treatment of Congenital Metabolic Diseases at our hospital launched the FIND project in collaboration with the MPS Association of Spain and with the endorsement of the Spanish Federation for Rare Diseases (FEDER) to identify possible cases of MPS and thus contribute to earlier diagnoses by increasing awareness of these diseases among pediatricians. The study protocol was approved by the Research Ethics Committee of Galicia (2013/390).

This nationwide selective screening project targeted an at-risk pediatric population, defined as patients whose pediatricians observed clinical signs and/or symptoms compatible with a diagnosis of MPS (Table 1). Participating pediatricians were sent a screening kit prepared at our hospital. The kit contained Whatman 903 analytical papers for the collection of blood and urine samples, an informed consent form to be signed by parents/guardians of the participating children, a short clinical guide on the warning signs and symptoms of MPS (Table 1) and on how to collect samples, a contact telephone number, and a postal address to which to the samples were to be sent.

**Table 1:**
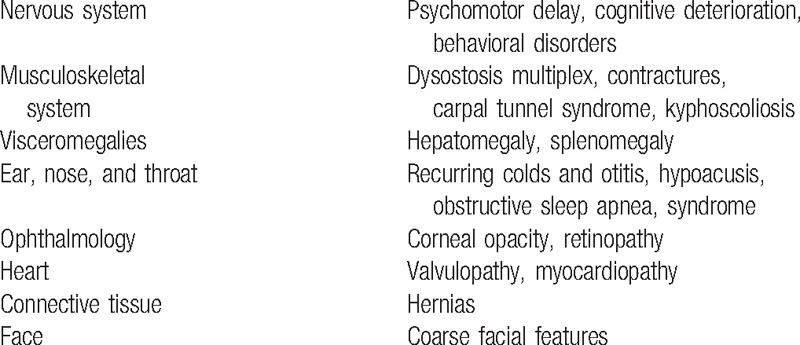
Warning signs and symptoms of mucopolysaccharidosis.

All pediatricians at primary care centers and hospitals in Spain were eligible to participate in the study. The program was disseminated with the support of MPS associations and families and the scientific societies involved. Pediatricians interested in participating contacted us and were sent a screening kit containing all the materials needed to collect the blood and urine samples. Patients with a positive initial screen underwent further clinical, laboratory, genetic, and enzyme studies.

### 2.2 Laboratory tests

#### 2.2.1 GAG determination

The GAG screening test was performed using a urine sample collected on Whatman 903 analytical paper. GAG levels were quantified using the dimethylmethylene blue (DMB) colorimetric assay,^[22]^ which is a simple, rapid, low-cost, and reliable method for quantifying urinary GAGs.^[23]^

To adjust absolute GAG levels to the concentration of the urine, urinary creatinine levels were simultaneously determined using the picric acid reaction, another classic colorimetric test.

To address potential sources of bias, all urine samples with a creatinine concentration of less than 20 mg/dL were considered diluted and all those with a concentration of more than 200 mg/dL were considered concentrated. In both cases, the final GAG concentration was considered inaccurate, and the corresponding pediatrician was notified and asked to send a new sample meeting the quality criteria.

GAG levels were referenced to creatinine levels using specific age-referenced values derived from a previous analysis of 145 urine samples from healthy children in different age groups (Table 2).

**Table 2:**
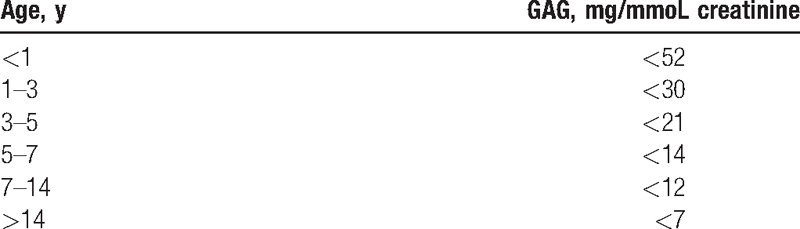
Normal urinary glycosaminoglycan (GAG) levels per age-specific group based on 145 samples from healthy children.

A liquid urine sample was requested in cases of high GAG levels. This sample was used to confirm and identify the GAGs present by electrophoresis in order to narrow the diagnosis (DS and HS for MPS I and II, HS for MPS III, KS and CS for MPS IVA and KS only for MPS IVB, DS for MPS VI, and DS, HS, and CS for MPS VII). Given that this technique detects the presence of GAGs through the binding of DMB and sulfate ions, it was not possible to identify MPS type IX because the substance accumulated is hyaluronic acid, which is devoid of sulfates.

#### 2.2.2 Enzyme determination

When a specific MPS was suspected, a 2nd analysis of the blood-impregnated paper was performed to determine the enzymatic activity of the following MPS by fluorometry: type I (alpha-iduronidase), type IIIB (N-acetyl-glucosaminidase), type IIIC (acetyl-CoA:α-glucosaminidase N-acetyltransferase), type IVA (galactose-6-sulfate sulfatase), type IVB (beta-galactosidase), type VI (arylsulfatase B), and type VII (beta-glucuronidase). Fluorometry is an analytical technique that involves adding a substrate marked with 4-methylumbelliferone. We followed the procedure described by Chamoles et al.^[24]^

Fluorometry was also used to quantify enzymatic activity for MPS types I, II, IIIC, IVA, IVB, VI, and VII in leukocytes and MPS IIIB in plasma.

#### 2.2.1 Genetic studies

A genetic study was not included in the selective screening test and was requested independently by each center as appropriate.

### 2.3 Statistical analysis

The size of the sample used to establish age-specific reference ranges for urinary GAG levels in healthy children was established to detect significant differences between healthy children and children with MPS (normal and pathological levels) using a 2-sided test, with a statistical power of 95% and a significance level of 5%.

Sensitivity, specificity, and positive predictive values were calculated using R software (R Core team 2016). To determine GAG cutoffs, we followed the NCCLS and Clinical and Laboratory Standards Institute (CLSI) guidelines C28-A2 and C28-A3 to estimate percentiles and 90% confidence intervals. In these guidelines, percentiles are calculated as observations corresponding to rank r = p∗(n + 1). We also followed the CLSI guidelines for the 90% confidence intervals of the reference limits; conservative confidence intervals were calculated using integer ranks (meaning that these intervals are at least 90% wide) https://www.medcalc.org/manual/referenceinterval.php.

## 3 Results

**Figure 1:**
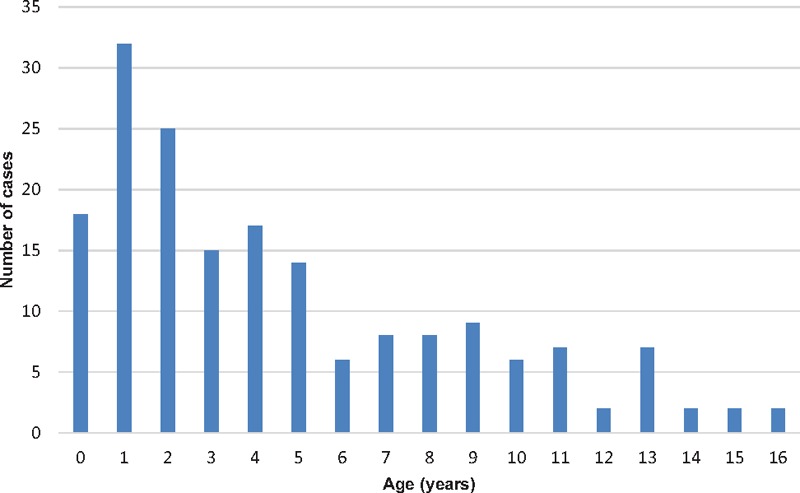
Urine and blood samples collected within the FIND project distributed by age.

A total of 389 kits were requested by pediatricians at hospitals (n = 238) and primary care centers or private practices (n = 151) in all regions of Spain between July 1, 2014 and June 30, 2016. Samples were received from 180 patients in 46 of the 50 provinces of Spain, and creatinine and GAG levels were determined in all cases. The age of these patients ranged from 0 to 16 years (mean, 4.7 ± 4.1 years; median, 3.5 years) (Fig. 1).

The samples were accompanied by short clinical reports in all cases. Most pediatricians reported just 1 or 2 signs and/or symptoms, mainly involving the nervous system, the musculoskeletal system, and the face (coarse features). In total, 571 different signs and symptoms were reported. Psychomotor delay with cognitive deterioration (17.3%) and behavioral disorders (9.3%) were the main neurological symptoms described. The main musculoskeletal manifestations reported were short stature (6.2%) and spinal involvement (7.4%). Recurrent otitis and colds accounted for 47.4% of otorhinolaryngological manifestations. Other important signs included visceromegalies (42 cases) and hernias (21 cases) (Table 3).

**Table 3:**
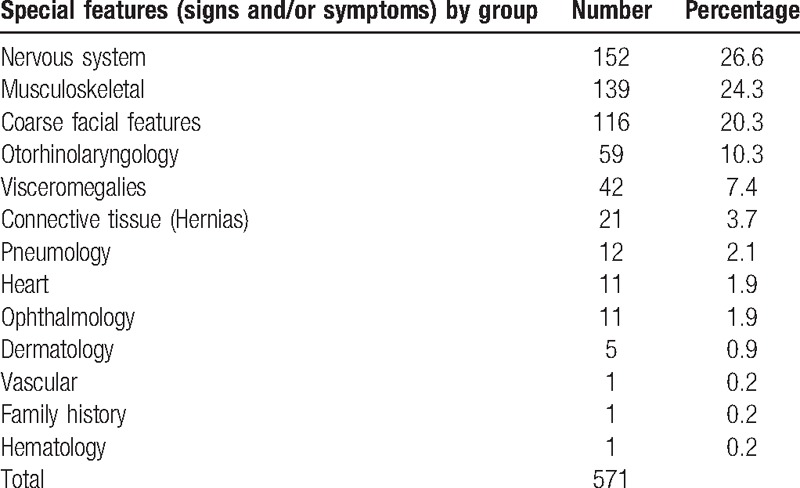
Clinical criteria reported by pediatricians who submitted the samples.

GAG levels were within the reference ranges in 147 of the 1st samples received (81.6%). A 2nd sample was requested in the remaining 33 cases (18.3%). In 21 samples, GAG levels were above the cutoff for the patients’ age, and in 12, creatinine levels were outside of the acceptable limits for a quality sample.

GAG levels were normal in 13 of the 33 cases in which a new sample was requested. In another 12 cases, total GAG levels were slightly elevated, but the electrophoresis study of the liquid urine sample was not compatible with any of the MPS types, so we suspect that there may have been interference from another substance excreted in the urine. Based on contact with the pediatricians who participated in the study, we learnt that 1 patient was diagnosed with mucolipidosis by massive parallel sequencing for the detection of lysosomal storage disorder mutations. Elevated GAG levels with low corresponding enzymatic activity were detected in 8 patients, all of whom were definitively diagnosed with MPS (Table 4). These 8 cases identified as a direct result of the selective screening strategy were counted as true positives because the sensitivity of our screening for MPS was 100% (85% specificity and 24% positive predictive value).

**Table 4:**
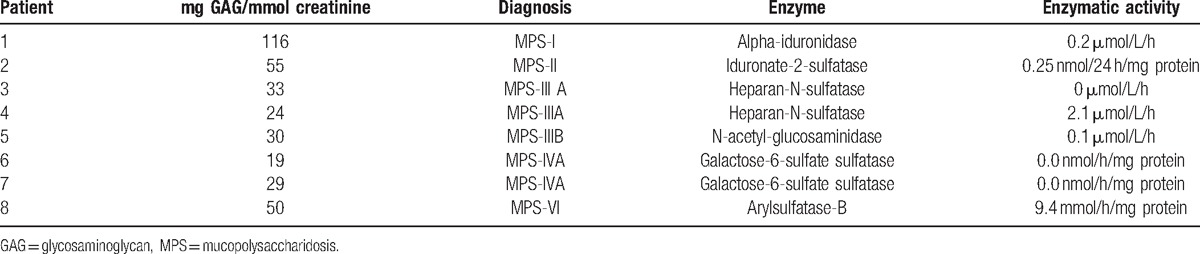
GAG levels and enzymatic activity in patients diagnosed with MPS within the FIND project.

In total, 75% of cases were detected in the 1st 3 years of life. The mean time from the presentation of clinical symptoms to detection was 22 months (range, 2–71 months), and only 2 cases the 1st year of starting the project were detected with 35 and 71 months of evolution of clinical symptoms. All 8 patients had clinical data compatible with a diagnosis of MPS. Seven had hepatomegaly and otorhinolaryngological and musculoskeletal symptoms, and 6 had coarse facial features. The 2 patients without coarse features were diagnosed with MPS VI and IVA. Connective tissue was affected in 4 cases and the central and/or peripheral nervous systems were affected in 5. Cardiac symptomatology was present in just 1 patient (MPS IVA) and ophthalmological symptomatology in 3 (Table 5).

**Table 5:**
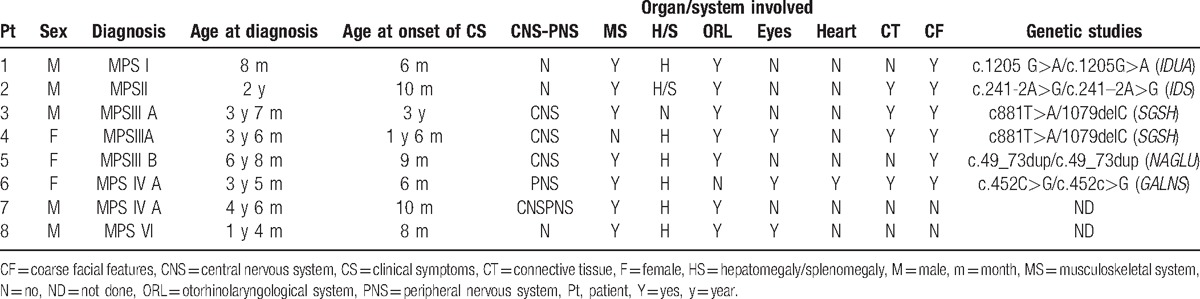
Characteristics of patients and data at diagnosis.

ERT was initiated in 5 of the 8 patients following diagnosis. The other 3 patients were diagnosed with MPSIII; 1 was included in the phase I/II trial of intrathecal enzymatic therapy for patients with MPS IIIB and another 2 will be included in the gene therapy trial for patients with MPS IIIA, which is predicted to begin in 2017.

## 4 Discussion

We have demonstrated that the detection of MPS at the onset of clinical symptoms is possible using a simple selective screening method. The method involves the fluorometric analysis of GAG levels using paper impregnated with urine and is applicable to all types of MPS except type IX. In addition, it is appropriate for population studies as the samples are easy to collect and remain stable at room temperature, meaning they can be sent through the post. Although other population studies of LSDs have used this method,^[25–27]^ to our knowledge, none has used it exclusively for the selective screening of MPS, with the exception of a recent validation study in this setting.^[28]^

The measurement of GAG levels in urine samples collected on analytical paper is simple and cost-effective. If levels are found to be elevated, the results are confirmed using a liquid urine sample to identify which GAGs are altered. In our case, we also performed an enzyme study because our center has the equipment needed to analyze blood samples on analytical paper. Fluorometric quantification of GAG levels costs less than 1 euro per sample and is much less expensive than other quantification methods requiring more complex technology.^[29,30]^ In addition, both urine and blood samples can be easily collected in an office setting with little or no pain. Finally, as already mentioned, the sample-impregnated papers can be sent by ordinary mail as GAGs remain stable for at least 20 days, even when subjected to large temperature changes.^[31]^ The above advantages make this selective screening method ideal for studies involving large, distant populations.

Following the results of this preliminary selective population screening study, we are currently performing a more detailed study of the false positive results observed in our series. Our initial results suggest that certain sulfate-containing medications used in the treatment of neurodegenerative lesions and/or the presence of other sulfated substances such as mucolipids may have an important role in such cases. Nonetheless, the study of the urine-impregnated papers had high specificity (85%) and high positive predictive value (24%) considering the low prevalence of MPS.^[2–6]^ A pilot study of an LSD screening program for newborns in Missouri, USA reported a positive predictive value of 14% for MPS.^[25]^ None of the patients with a false negative result in our series were diagnosed with MPS using other methods, and therefore we can provisionally report a negative predictive value and sensitivity of 100%.

Our study has several strengths, including the establishment of age-specific reference values for GAG levels in our population, its prospective design, and follow-up period of 2 years. In addition, measurement of GAGs by tandem mass spectrometry combines the advantages of improved selectivity and excellent sensitivity with multiplexing capability.^[30]^ Several limitations, however, should be acknowledged. First, DMB-based spectrophotometry can give rise to false negatives, especially in MPS III and IV.^[32,33]^ Second, low levels of GAG may be excreted in the urine. Tomatsu et al^[32,33]^ described possible cases of false negatives in adult MPS IVA, especially in patients with milder forms who may not excrete KS in the urine, preventing thus detection by DMB. Finally, elevated GAG levels may be due to conditions unrelated to MPS, such as diabetes, arthritis, or mucolipidosis (as occurred in our study).^[34,35]^

The selective screening method within the FIND project described in this study identified 8 hitherto undiagnosed cases of MPS (I, II, III, IV, and VI) in patients with clear signs and symptoms. The most common manifestations were hepatomegaly, otorhinolaryngological and musculoskeletal symptoms, and coarse facial features. Twenty-five percent of the cases were identified in patients at 1 year of age and 75% were detected within the 1st 3 years of life, highlighting the potential usefulness of selective symptom-based screening for the early diagnosis of MPS. The time from onset of clinical manifestations to detection of 22 months is shorter than that reported in the literature,^[8,9]^ which leads us to believe that the effectiveness of this screening method would be greatly improved by adequate education and training of pediatricians. Scientific advances have led to a steady increase in the treatments available for MPS.^[20,36–39]^ In fact, 5 of the 8 patients diagnosed in our series have already begun to receive ERT following international standards, and the other 3 are involved in phase I/II clinical trials.

In summary, the FIND project is a useful, cost-effective tool for the early detection of MPS and may be a valuable 1st-tier screening test for high-risk groups.
